# Serum Isthmin-1 levels are positively correlated with macrovascular complications in type 2 diabetic patients

**DOI:** 10.3389/fendo.2025.1594158

**Published:** 2025-06-26

**Authors:** Yajing Wang, Yingjie Feng, Xue Wang, Xicui Zong, Jing Liu, Shujin Liang, Yiqun Meng, Penghua Fang, Nianlan Zhou, Na Luo, Zhenwen Zhang

**Affiliations:** ^1^ Department of Endocrinology, Northern Jiangsu People’s Hospital Affiliated to Yangzhou University, Yangzhou, China; ^2^ Department of Endocrinology, Northern Jiangsu People’s Hospital, Yangzhou, China; ^3^ Laboratory Training Center, Nanjing University of Chinese Medicine Hanlin College, Taizhou, Jiangsu, China; ^4^ Key Laboratory for Metabolic Diseases in Chinese Medicine, First College of Clinical Medicine, Nanjing University of Chinese Medicine, Nanjing, China; ^5^ Department of Gastroenterology, Northern Jiangsu People’s Hospital Affiliated to Yangzhou University, Yangzhou, China

**Keywords:** Isthmin-1, macrovascular complications of diabetes, T2DM, glucose metabolism, lipid metabolism

## Abstract

**Objective:**

Isthmin-1 (ISM-1), a novel adipokine, has dual effects of increasing fat and glucose uptake while inhibiting hepatic fat synthesis. However, little literature has been found dealing with ISM-1 levels in type 2 diabetes patients with macroangiopathy. The aim of the study was to evaluate possible relationships between ISM-1 peptide levels and macrovascular (MACV) complications in type 2 diabetic subjects.

**Methods:**

The study groups consisted of 20 normal controls (NC), 20 T2DM subjects, and 65 MACV subjects. Serum ISM-1 concentrations were determined using immunosorbent assay kits. Linear regression analysis was used to assess the correlation between serum ISM-1 levels and glucose and lipid indicators.

**Results:**

The results showed that the serum ISM-1 levels were higher in T2DM subjects than normal controls but lower than MACV subjects (1.20 (0.86, 1.83) vs. 2.07 (1.06, 4.09), P<0.0001). In addition, positive correlations were found between: ISM-1 and systolic blood pressure (SBP) (r = 0.2934; P= 0.0024), ISM-1 and diastolic blood pressure (DBP) (r = 0.2041; P= 0.0368), ISM-1 and triglyceride (TG) (r = 0.3388; P= 0.0004), ISM-1 and FBG (r = 0.2586; P= 0.0077), ISM-1 and HbA1c (r = 0.4002; P< 0.0001), ISM-1 and TyG index (r = 0.3342; P= 0.0005), ISM-1 and HOMA-IR (r = 0.2558; P = 0.0085) in both MACV, T2DM and normal control subjects. Negative correlations were found between ISM-1 and high-density lipoprotein cholesterol (HDL-C) (r = -0.4065; P< 0.0001) and ISM-1 and HOMA-IS (r = -0.2106; P = 0.0319) in both subjects.

**Conclusions:**

Our results indicated that MACV individuals have higher serum ISM-1 levels, and ISM-1 was positively correlative to glucolipid metabolism and blood pressure, suggesting ISM-1 may participate in the occurrence and development of MACV mainly by affecting glucose and lipid metabolism

## 1 Introduction

By 2045, IDF projections show that 1 in 8 adults, approximately 783 million, will be living with diabetes, an increase of 46%. With the increasing incidence of diabetes worldwide, this chronic disease has become one of the important challenges affecting human health and quality of life. The characterization of diabetes mellitus, especially T2DM, is hyperglycemia and insulin resistance (1). Excess blood glucose can participate in multiple harmful metabolic pathways leading to the production of ceramides and excessive reactive oxygen species (ROS). These products can activate signaling molecules associated with diabetic macroangiopathy, such as protein kinase C, ultimately contributing to the occurrence of diabetic macroangiopathy. Besides, the disorder of lipid metabolism related to T2DM can result in excessive lipid deposition and subsequent lipotoxicity, which contributes to the occurrence of diabetic macroangiopathy. Therefore, understanding the precise molecular mechanism by which pathogenic variables cause diabetic macroangiopathy will facilitate the development of innovative therapies to combat the condition.

ISM-1, as an important adipokine discovered in recent years, plays a key role in physiological processes such as cell metabolism, energy balance and insulin sensitivity (2, 3). Many studies have shown that the abnormal expression or function of ISM-1 is closely related to the occurrence and development of diabetes and its complications. Previous clinical research has indicated that serum ISM-1 level is significantly increased in T2DM patients with diabetic retinopathy (DR) and is positively correlated with the risk of DR. It is an independent risk factor for DR in patients with type 2 diabetes mellitus (4). In addition, serum ISM-1 levels were positively and independently associated with the severity of albuminuria in patients with type 2 diabetes (5). Moreover, serum ISM-1 is negatively correlated with HDL-C in patients with T2DM (6). However, the relationship between serum ISM-1 functions and MACV in patients with T2DM has not been studied. Therefore, the aim of this study was to investigate the relationship between serum ISM-1 levels and MACV in patients with T2DM.

## 2 Materials and methods

### 2.1 Study design and participants

The present study was conducted at the Clinical Medical College, Yangzhou University. The present study consisted of 20 healthy volunteers, 20 T2DM volunteers, and 65 MACV volunteers, healthy volunteers according to a physical examination and routine laboratory tests. In this study, each participant gave official written consent, had normal exercise and eating behavior, and had no fat preference or fat aversion. Individuals with active hepatitis or liver cirrhosis, chronic renal failure on hemodialysis, or other known major diseases were precluded from the study. Diabetic ketoacidosis or hyperglycemic hyperosmolar state was excluded. To ensure the accuracy of the diagnosis of T2DM, we excluded patients with a disease duration of <1 month. To ensure the authenticity of blood lipid measurements, patients are using lipid-lowering medications. The assignment of patients with MACV, T2DM and normal controls must meet the diagnostic criteria of the World Health Organization. For T2DM: fasting venous serum value: ≥126 mg/dl or 75 g (2h) oral glucose tolerance test (OGTT) venous serum value: ≥200 mg/dl (7). Patients without typical symptoms need to be retested on different days. For normal controls: fasting venous serum value: <100 mg/dl or 75 g (2-h) OGTT venous serum value: <140 mg/dl (7). For MACV: The patients met the diagnostic criteria of cerebral atherosclerosis, carotid artery disease, coronary heart disease, or lower extremity atherosclerosis. The protocol of this study was approved by the Ethics Committee of Northern Jiangsu People’s Hospital Affiliated to Yangzhou University (No. 2022ky171-2).

### 2.2 Sample collection and detection

After an overnight fast, blood samples were collected from each participant at 8:00 a.m. and immediately centrifuged (4°C) as described previously. Briefly, within 30 minutes of collection, the blood samples (2 mL) were placed in prechilled EDTA tubes containing 100 μl of aprotinin (1 μg/mL) and centrifuged for 15 min. at 1000 g at 4°C. The separated serum was placed into vials and kept at -80°C until it was measured. Serum Ism-1 levels were measured using commercially available ELISA kits (Jianglaibio, Shanghai (China); JL14441). According to the manufacturer’s specification, the assay range for ISM-1 was 0.156–10 ng/mL, intra-assay precision CV%<10%, and inter-assay precision CV%<10%. The mean of the two measurements, which were all taken in duplicate, was taken into account. Age, sex, height, weight, blood pressure (SBP and DBP), history of smoking, alcohol consumption, lipid-lowering drug application and history of hypertension were assessed at enrollment. Clinical data were obtained from the hospital electronic medical record system and included HbA1c, total cholesterol (TC), triglyceride (TG), low-density lipoprotein cholesterol (LDL-C) and high-density lipoprotein cholesterol (HDL-C). Weight and height data were used to calculate BMI (BMI = weight (kg)/height (m2)). The HOMA-IR Index was calculated for each participant using the formula [fasting glucose (mmol/L) × fasting insulin (mIU/L)/22.5]. The HOMA-β (INS) index was calculated as [20 × fasting insulin (mIU/L)/(fasting glucose (mmol/L)-3.5) (%)]. The HOMA-IS index was calculated using the formula [1/HOMA-IR]. The HOMA-CR index was calculated as [1.5+fasting glucose (mmol/L) × fasting C peptide (ng/ml)/(2.8×0.333)]. The HOMA-β (C-peptide) index was calculated as [270×fasting C-peptide (ng/ml)/(fasting glucose (mmol/L)-3.5) (%)].

## 3 Statistical analysis

The statistical analyses were performed with GraphPad Prism v6.0 (GraphPad Software). All data were presented as mean ± SD. The differences between the groups were analyzed with an independent t-test or a Mann-Whitney test in the non-parametric distributions (not normally distributed). Possible correlations between parameters were evaluated by Pearson’s correlation coefficient analyses (normally distributed) or Spearman’s correlation coefficient analyses (not normally distributed). Statistical significance was considered to be P< 0.05.

## 4 Results

### 3.1 Serum ISM-1 levels were different in T2DM patients with or without MACV

**Figure 1:**
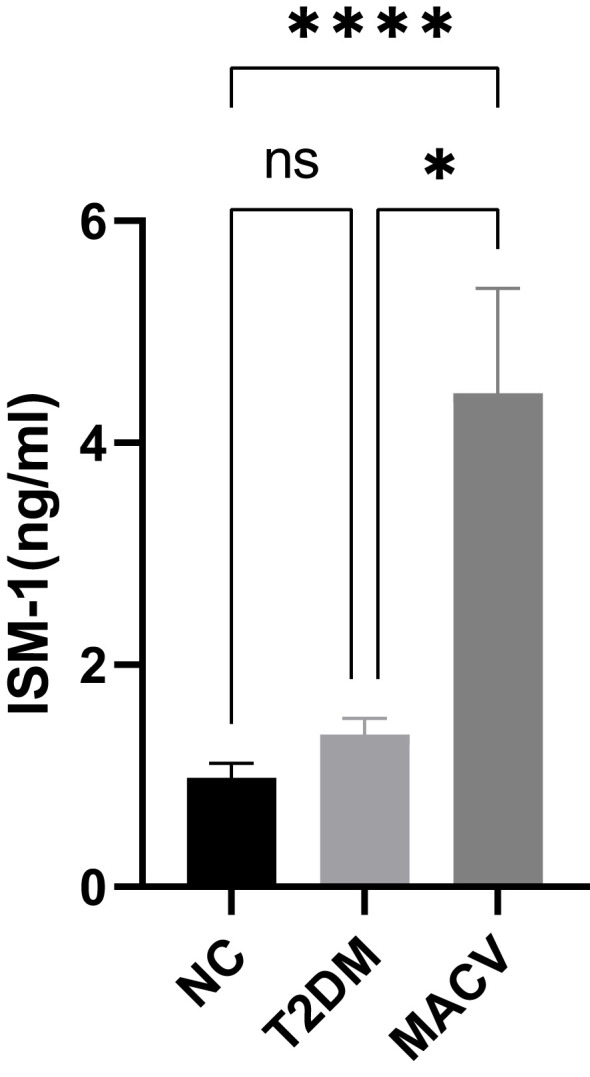
Comparison of the levels of serum ISM-1 in different groups. Data are represented as mean ± SEM. ****P < 0.0001; *P<0.05; ns, no signifcance. ISM- 1, isthmin- 1.

The main indexes of sex, age, duration of diabetes, body height, body weight, and BMI in the three groups are listed in **Table 1**. Except for the course of disease, which was observed as the difference between the NC group and the other groups, there were no statistically significant differences in other baseline indicators among the three groups. Compared with the T2DM group, the MACV group had a higher ISM-1 level (see **Figure 1**), but no difference was observed between the NC group and the T2DM group (p > 0.05). The MACV group also had a higher level of ISM-1 than the NC group and was statistically significant.

**Table 1:** Patient baseline characteristics.

Characteristics	NC (n=20)	T2DM (n=20)	MACV (n=65)	*P* value
Sex (male/female)	20(5/15)	20(10/10)	65(33/32)	0.118
Age (year)	49.85 ± 10.65	49.60 ± 14.71	51.22 ± 9.16	0.784
Duration of diabetes (year)	-	9.5 (6,12)	10(2.5,15)	-
Height(cm)	167.55 ± 7.04	167.50 ± 7.93	166.42 ± 7.13	0.750
Weight(kg)	62.69 ± 9.42	65.43 ± 9.66	64.29 ± 12.53	0.750
BMI (kg/m2)	22.28 ± 2.62	23.28 ± 2.87	23.10 ± 3.45	0.544

### 3.2 Association of serum ISM-1 levels with lipid metabolism in T2DM patients

**Figure 2:**
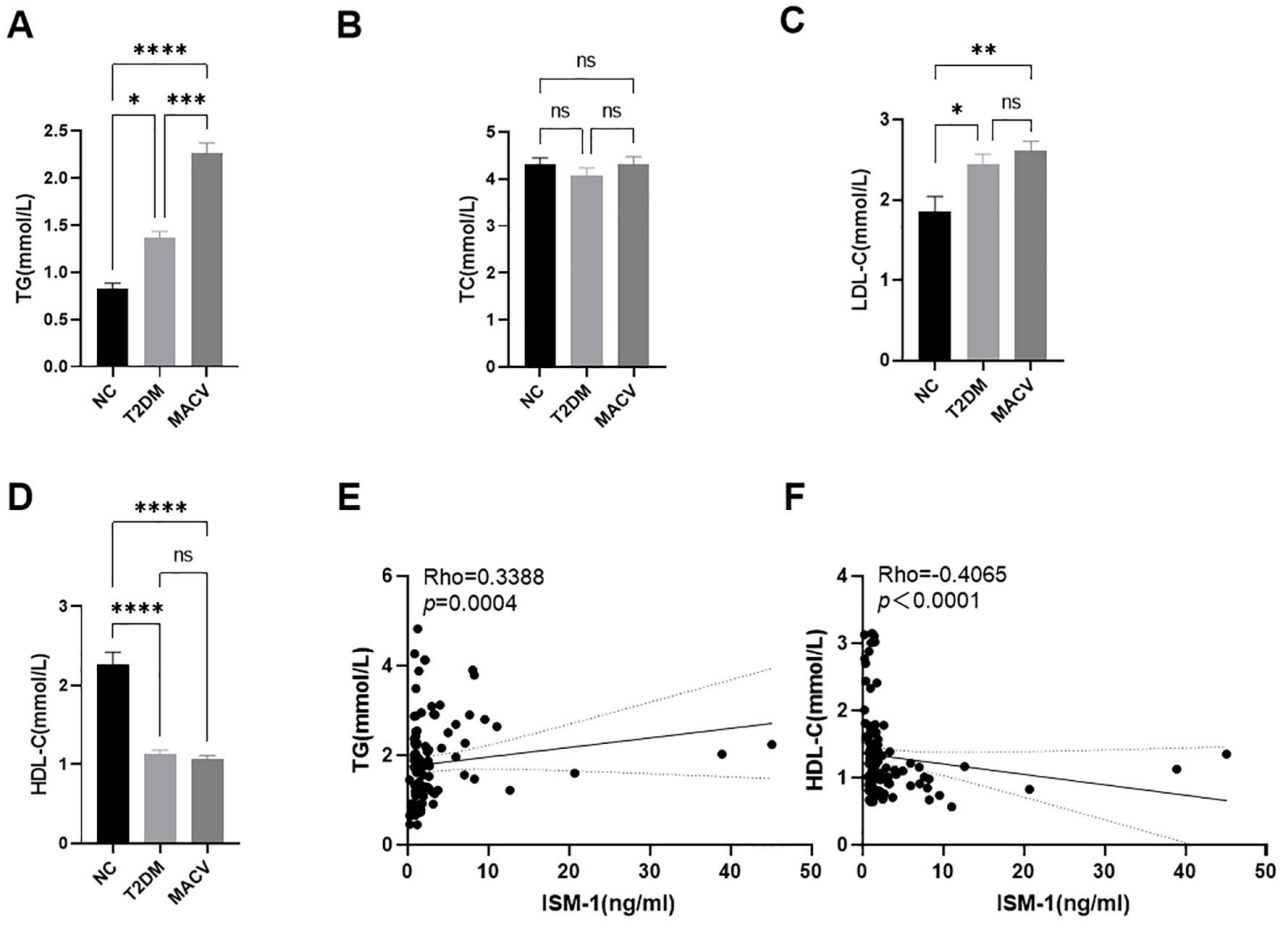
Comparison of the levels of lipid metabolism related indicators in different groups, simple linear regression of serum Ism-1 levels and TG, HDL-C. **(A–D)** The levels of TG, TC, LDL-C and HDL-C in different groups. **(E, F)** The simple linear regression of serum Ism-1 levels and TG, HDL-C. Data are represented as mean ± SEM. ****P < 0.0001; ***P < 0.001; **P < 0.01; *P < 0.05; ns, no signifcance.

Due to disorders of lipid metabolism playing an important role in the pathogenesis of type 2 diabetes mellitus and macrovascular disease, we performed a comparison and correlation between ISM-1 and lipid metabolism indices between groups (**Figure 2**). The study found that compared with the NC group, the triglyceride and low-density lipoprotein in the T2DM group and MACV group increased significantly, and the high-density lipoprotein decreased significantly. Compared with the T2DM group, the triglyceride in the MACV group increased significantly. Further correlation analysis showed that triglyceride was positively correlated with ISM-1, while high-density lipoprotein was negatively correlated with ISM-1.

### 3.3 Association of serum ISM-1 levels with glucometabolic and insulin resistance in T2DM patients

**Figure 3:**
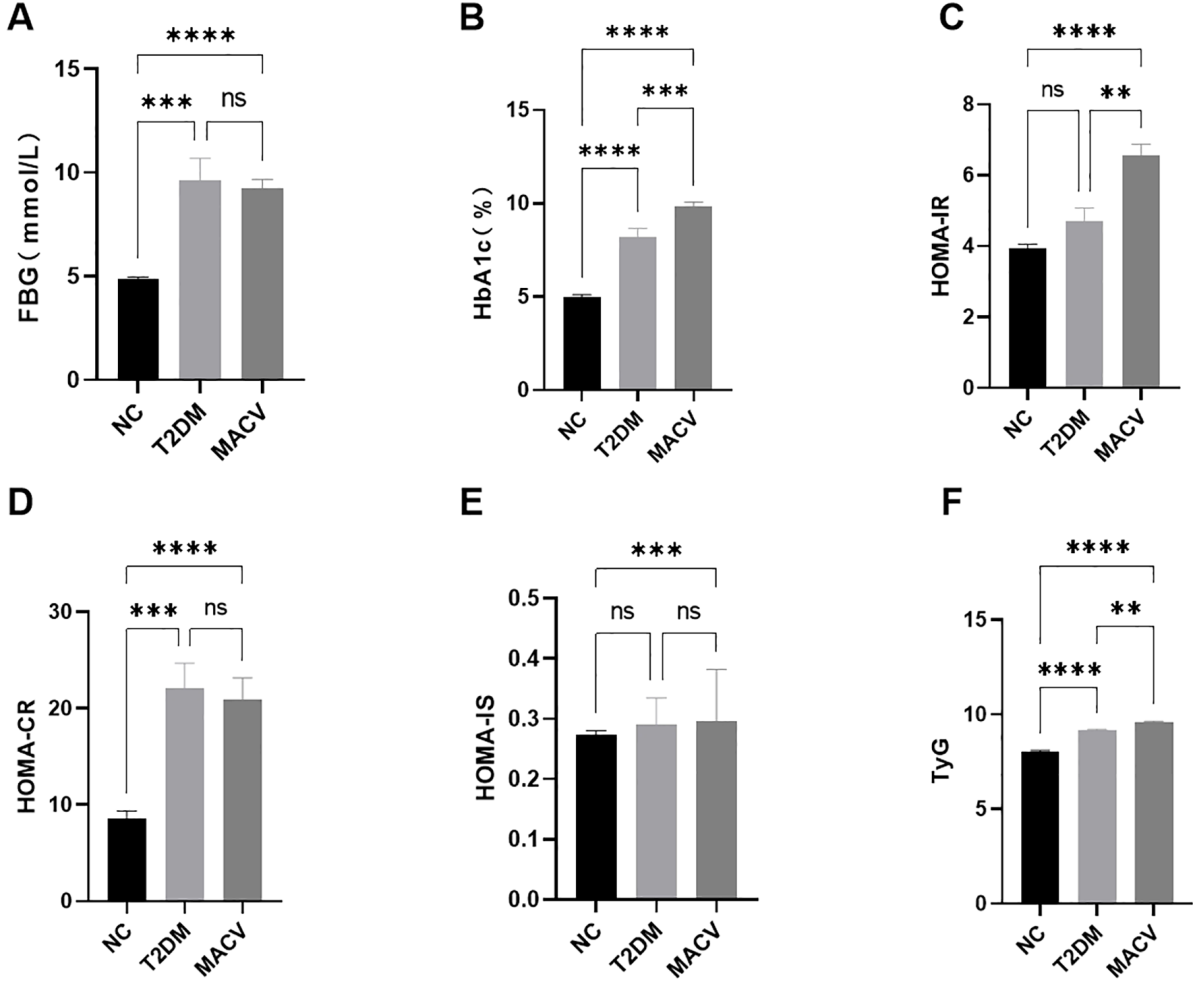
Comparison of the levels of glycometabolism related indicators in different groups. **(A–F)** The levels of FBG, HbA1c, HOMA-IR, HOMA-CR, HOMA-IS and TyG index in different groups. Data are represented as mean ± SEM. ****P < 0.0001; ***P < 0.001; **P < 0.01; ns, no signifcance.

**Figure 4:**
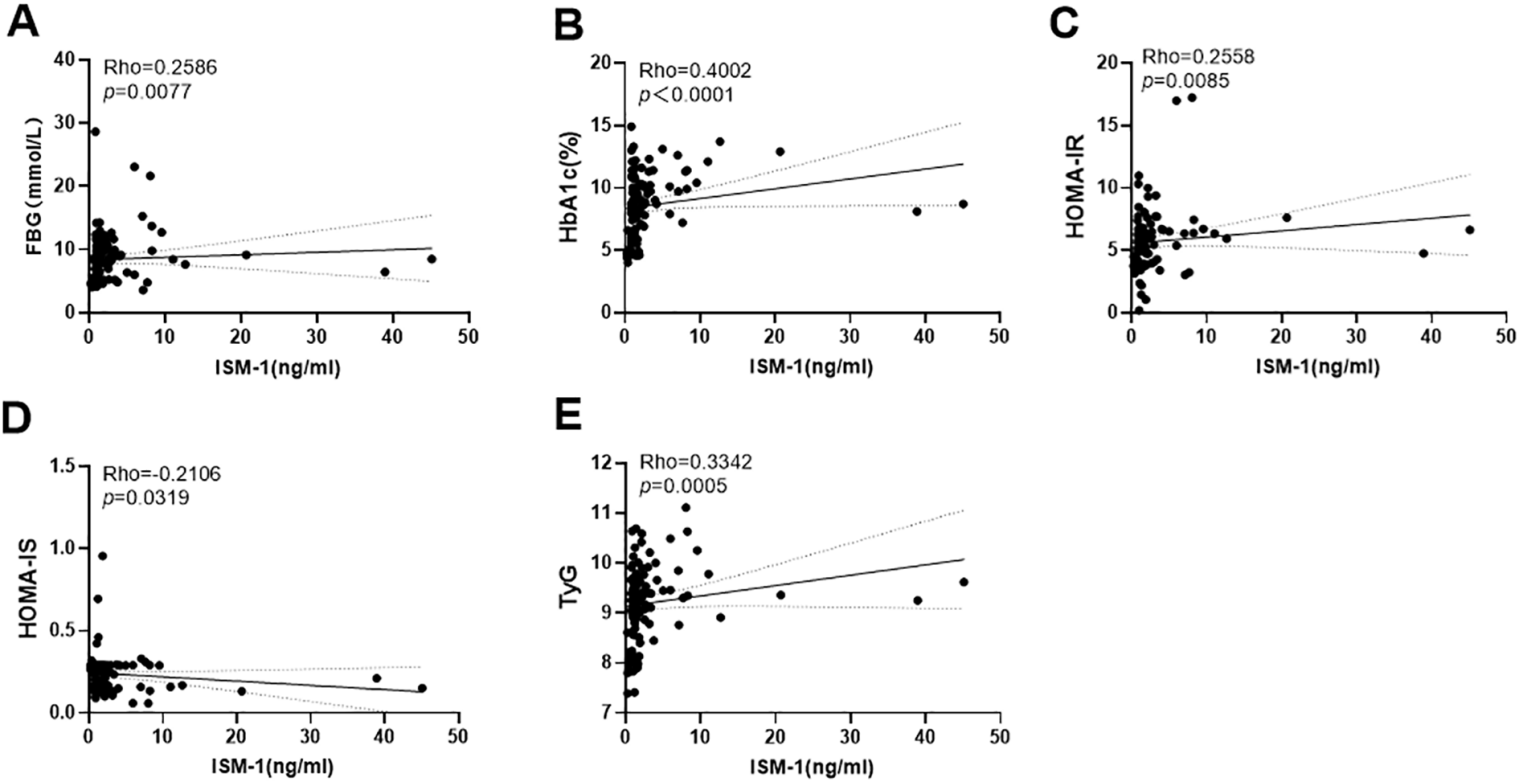
Simple linear regression of serum Ism-1 levels and FBG, HbA1c, HOMA-IR, HOMA-IS and TyG **(A–E)**.

To investigate whether serum ISM-1 levels were associated with insulin resistance, we performed group comparisons (**Figure 3**) and analysis of the correlation between ISM-1 and indicators of glucose metabolism (**Figure 4**). The study found that fasting blood glucose, glycosylated hemoglobin (HbA1c), HOMA-CR, and TyG were significantly increased in the T2DM group and the MACV group compared with the NC group. Compared with T2DM group, HbA1c, HOMA-IR, and TyG were significantly increased in MACV group. Correlation analysis showed that FBG, HbA1c, HOMA-IR, and TyG were positively correlated with ISM-1, while HOMA-IS was negatively correlated with ISM-1.

### 3.4 Association of serum ISM-1 levels with vascular function in T2DM patients

**Figure 5:**
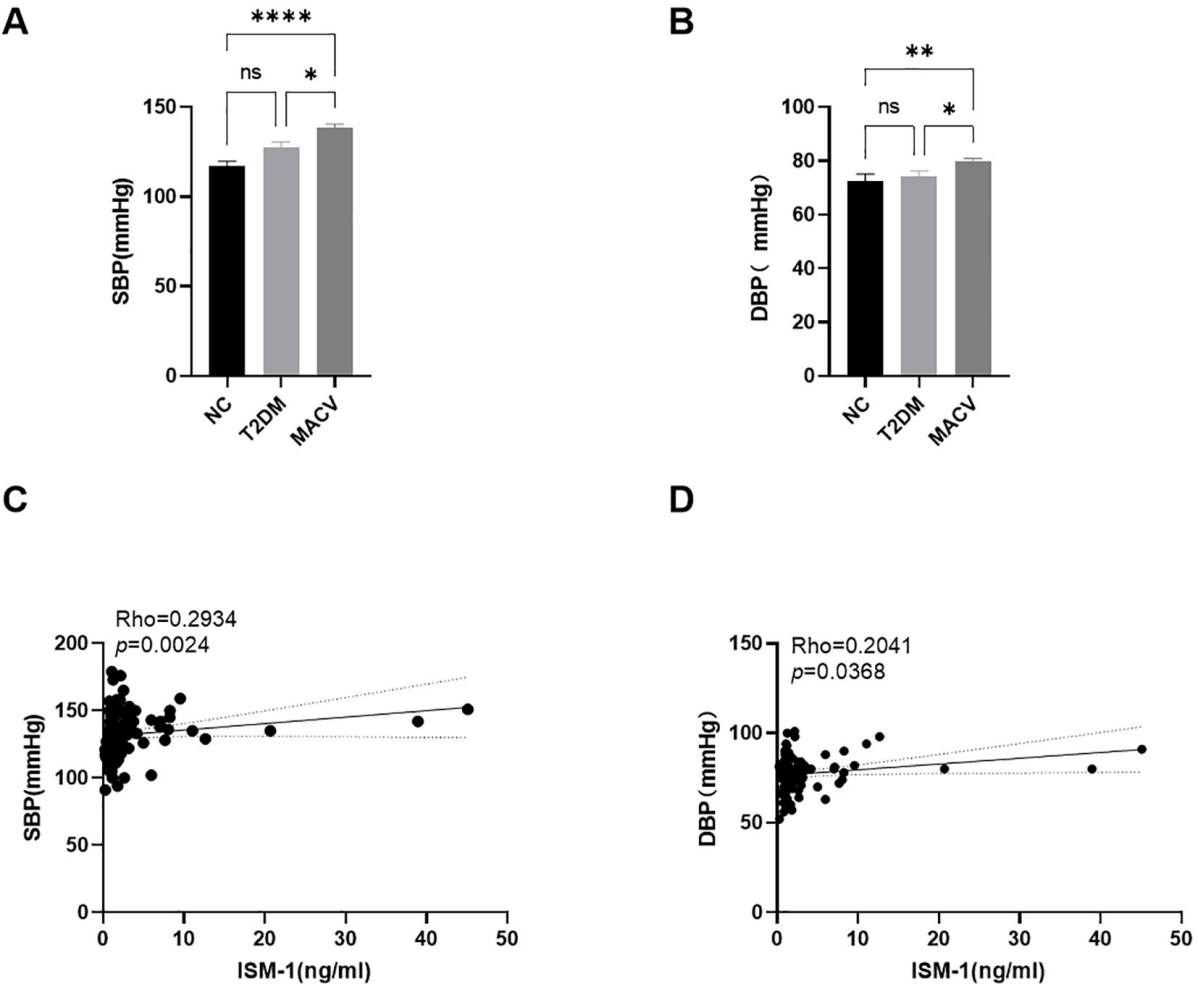
Comparison of the levels of vascular function related indicators in different groups, simple linear regression of serum Ism-1 levels and SBP, DBP. **(A, B)** The levels of SBP and DBP in different groups. **(C, D)** The simple linear regression of serum Ism-1 levels and SBP, DBP. Data are represented as mean ± SEM. ****P < 0.0001; **P < 0.01; *P < 0.05; ns, no signifcance.

We further assessed the relationship between vascular function and ISM-1 levels by intergroup comparison and correlation analysis (**Figure 5**). The results showed a significant decrease in both systolic (SBP) and diastolic blood pressure (DBP) in the NC and T2DM groups compared to the MACV group. Moreover, both SBP and DBP were positively correlated with ISM-1.

## 5 Discussion

ISM-1 was first discovered in 2002 by Pera in the Isthmin brain of Xenopus laevis, hence the name Isthmin (8). Subsequently, it was gradually found that it was widely present in brain, lung, eye, ear, and other tissues with dynamic changes and participated in the regulation of growth and development, cell metabolism, tissue reconstruction, immune response and other processes through endocrine mechanisms (3, 9, 10). To our best knowledge, there are no other data concerning ISM-1 concentrations in type 2 diabetes with macrovascular complications. The present study shows that a statistically significant higher level of ISM-1 was found in T2DM with MACV compared with T2DM. Also, a positive correlation was found between ISM-1 and SBP, DBP, TG, FBG, HbA1c, HOMA-IR, as well as TyG index in both MACV, T2DM, and normal control subjects.

It has been found that frequent blood glucose fluctuations lead to increased oxidative stress, which damages the vascular endothelium and promotes the development of atherosclerosis, while it may also affect vasodilatation, leading to an increased risk of cardiovascular events (11). This is corroborated by the fact that HbA1c levels and HOMA-IR showed significant differences between all groups in this study. It is worth noting recently that ISM-1 has been found to be closely related to the occurrence and development of microvascular complications. Previous studies have shown that serum levels of ISM-1 in DKD patients are significantly higher than those in T2DM patients without DKD (12). In T2DM patients with DR, ISM-1 also influences retinal blood vessels to participate in the occurrence and development of DR by mediating the increase of endothelial permeability (4). Consistent with these results, our study revealed that circulating ISM-1 levels were higher in MACV patients compared to the T2DM group, suggesting that ISM-1 is closely related to MACV and may be an independent risk factor for MACV. However, there was no significant difference in the level of ISM-1 between the NC group and the T2DM group. This may also be due to the fact that ISM-1 expression was positively correlated with BMI but not with glucose levels (13). The upregulation of ISM-1 in overweight T2DM patients may be more obvious, but there was no significant statistical significance in the BMI of members between groups in this study. Furthermore, FBG, HbA1c, HOMA-IR and TyG were also positively correlated with ISM-1, while HOMA-IS was negatively correlated with ISM-1. These data seem to indicate that the upregulation of ISM-1 may be a consequence of elevated glucose. Recent studies demonstrate that ISM-1 can activate the PI3K-AKT signal transduction pathway to promote glucose uptake independent of insulin and insulin-like growth factor receptor (14). Besides, it has also been reported that ISM-1 indirectly promotes pancreatic β cell dysfunction through NODAL (a member of the transforming growth factor superfamily), thereby accelerating the transition from pre-diabetes to type 2 diabetes (15). These results suggested that the higher level of ISM-1 observed in MACV patients may represent an adaptation to the increase of insulin resistance associated with MACV in T2DM because the levels for HOMA-IR changed significantly between the T2DM group and the MACV group in this study.

The TyG index is a comprehensive index that assesses the volume, density and distribution of fat in patients with T2DM, reflecting levels of both glucose metabolism and lipid metabolism (16). Distinguishing the TyG index from other insulin resistance indices, it was significantly different between the groups, suggesting that lipid metabolism may play an important role in the pathogenesis of MACV in patients with type 2 diabetes mellitus. As an adipokine, ISM-1 has been shown to play an important role in the regulation of lipid metabolism. ISM-1 is able to counteract lipid accumulation in the liver by converting liver cells from a lipid-producing state to a protein synthesis state. In addition, therapeutic doses of recombinant ISM-1 were able to improve liver steatosis in diet-induced fatty liver mouse models (3). ISM-1 can also inhibit insulin-regulated lipid synthesis, promote protein synthesis, and affect human lipid metabolism and protein metabolism in the non-insulin pathway (17). In this study, we also found that the TG was positively correlated with ISM-1, while HDL-C was negatively correlated with ISM-1. This may be related to the effect of ISM-1 on glucose uptake by adipocytes, which in turn may regulate lipid metabolism by affecting intracellular energy metabolism and lipid synthesis pathways. HDL-C plays an important role in cholesterol transport and metabolism and can play a protective role in the occurrence and development of MACV by reducing the deposition of LDL-C in the blood vessel wall, alleviating oxidative stress and inflammation in the blood vessel wall (18). Besides, a number of studies have shown that the TyG index performs well in evaluating and predicting the risk of MACV in T2DM patients, and the mechanism may involve pathophysiological processes such as inflammation and oxidative stress (19, 20). Combined with the results of our study, ISM-1 has the potential to influence the pathogenesis of diabetic macrovascular disease by affecting HDL and lipid metabolic processes. Moreover, both SBP and DBP levels increased significantly in the MACV group but not in the T2DM group compared with the NC group. The levels of SBP and DBP, as the most basic and direct indicators of vascular compliance and elasticity, are strongly correlated with vascular compliance and elasticity (21). Correlation analysis in this study revealed that ISM-1 was positively correlated with SPB and DBP. This further confirms that ISM-1 may be one of the indirect indicators for the assessment of vascular function in patients with type 2 diabetes mellitus.

In summary, we found that serum ISM-1 level is correlated with MACV in type 2 diabetes patients for the first time, and there is a significant correlation with HOMA-IR, HDL-C, TG, TyG index, and other indicators. ISM-1 may participate in the occurrence and development of MACV mainly by affecting glucose and lipid metabolism, but the specific causal relationship and specific mechanism between the two remain to be further studied. Besides, as a cross-sectional study, it is unclear whether serum levels of ISM-1 change with the exacerbation of MACV. Finally, due to the limited sample size, the relationship between different types of MACV and the level of ISM-1 needs to be further studied after expanding the sample size.

## Data Availability

The original contributions presented in the study are included in the article/supplementary material. Further inquiries can be directed to the corresponding author/s.
